# 3-(2-Bromo-4,5-dimethoxy­phen­yl)propiononitrile

**DOI:** 10.1107/S1600536808013767

**Published:** 2008-05-14

**Authors:** Yan-Ping Liu, De-Cai Wang, Hui Chen, Si-Shun Kang, Xin-Ming Huang

**Affiliations:** a State Key Laboratory of Materials-Oriented Chemical Engineering, College of Life Sciences and Pharmaceutical Engineering, Nanjing University of Technolgy, Xinmofan Road No. 5 Nanjing, Nanjing 210009, People’s Republic of China; b College of Science, Nanjing University of Technolgy, Xinmofan Road No. 5 Nanjing, Nanjing 210009, People’s Republic of China

## Abstract

In the mol­ecule of the title compound, C_11_H_12_BrNO_2_, a weak intra­molecular C—H⋯Br hydrogen bond results in the formation of a five-membered ring, which adopts an envelope conformation with the H atom displaced by 0.486 Å from the plane of the other ring atoms. In the crystal structure, inter­molecular C—H⋯O hydrogen bonds link the mol­ecules.

## Related literature

For related literature, see: Kametani *et al.* (1973 ▶); Paull & Cheng (1972 ▶); Lerestif *et al.* (2005 ▶).
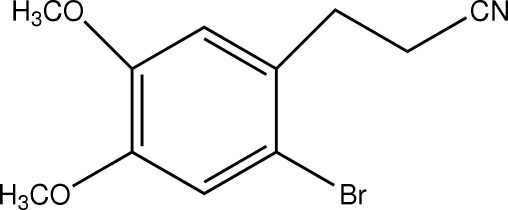


## Experimental

### 

#### Crystal data

C_11_H_12_BrNO_2_
*M* *_r_* = 270.13
Tetragonal, 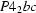

*a* = 17.552 (3) Å
*c* = 7.4870 (15) Å
*V* = 2306.5 (7) Å^3^
*Z* = 8
Mo *K*α radiation
μ = 3.54 mm^−1^
*T* = 294 (2) K
0.30 × 0.10 × 0.10 mm

#### Data collection

Enraf–Nonius CAD-4 diffractometer
Absorption correction: ψ scan (North *et al.*, 1968 ▶) *T*_min_ = 0.416, *T*_max_ = 0.718
4281 measured reflections
1128 independent reflections
657 reflections with *I* > 2σ(*I*)
*R*_int_ = 0.047
3 standard reflections frequency: 120 min intensity decay: none

#### Refinement

*R*[*F* ^2^ > 2σ(*F* ^2^)] = 0.052
*wR*(*F* ^2^) = 0.113
*S* = 0.99
1128 reflections
137 parameters
H-atom parameters constrained
Δρ_max_ = 0.36 e Å^−3^
Δρ_min_ = −0.40 e Å^−3^
Absolute structure: Flack (1983 ▶), with no Friedel pairs
Flack parameter: 0.00 (3)

### 

Data collection: *CAD-4 Software* (Enraf–Nonius, 1989 ▶); cell refinement: *CAD-4 Software*; data reduction: *XCAD4* (Harms & Wocadlo, 1995 ▶); program(s) used to solve structure: *SHELXS97* (Sheldrick, 2008 ▶); program(s) used to refine structure: *SHELXL97* (Sheldrick, 2008 ▶); molecular graphics: *PLATON* (Spek, 2003 ▶); software used to prepare material for publication: *SHELXTL* (Sheldrick, 2008 ▶).

## Supplementary Material

Crystal structure: contains datablocks global, I. DOI: 10.1107/S1600536808013767/hk2461sup1.cif

Structure factors: contains datablocks I. DOI: 10.1107/S1600536808013767/hk2461Isup2.hkl

Additional supplementary materials:  crystallographic information; 3D view; checkCIF report

## Figures and Tables

**Table 1:** Hydrogen-bond geometry (Å, °)

*D*—H⋯*A*	*D*—H	H⋯*A*	*D*⋯*A*	*D*—H⋯*A*
C2—H2*A*⋯O1^i^	0.97	2.32	3.193 (10)	150
C3—H3*B*⋯Br	0.97	2.76	3.195 (9)	108

Symmetry code: (i) 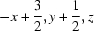.

**Fig. 1.**
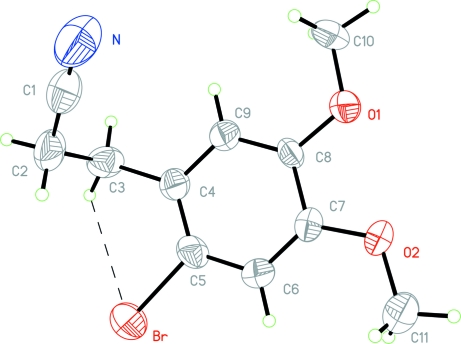
The molecular structure of the title molecule, with the atom-numbering scheme. Displacement ellipsoids are drawn at the 30% probability level. Hydrogen bond is shown as dashed line.

**Fig. 2.**
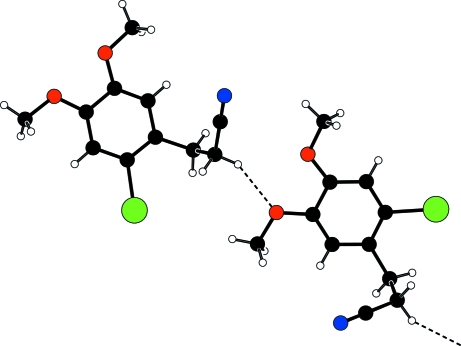
A partial packing diagram of (I). Hydrogen bonds are shown as dashed lines.

## supplementary crystallographic information

### Comment

2-Bromo-4,5-dimethoxyhydrocinnamonitrile is the precursor of 1-cyano-4,5-di- methoxybenzocyclobutene, which is a key intermediate of ivabradine (Lerestif *et al.*, 2005), xylopinine (Kametani *et al.*, 1973) and 4-substituted 3a,4,5,9 b-terahydrobenz[*e*]isoindolinea (Paull & Cheng, 1972). As part of our studies in this area, we report herein the synthesis and crystal structure of the title compound, (I).

In the molecule of (I), (Fig. 1), ring A (C4-C9) is, of course, planar. Br, O1, O2, C3 and C10 atoms lie in the ring plane. A weak intramolecular C-H···Br [C3-H3B = 0.97, H3B···Br = 2.76, C3···Br = 3.195 (9) Å and C3-H3B···Br = 108°] hydrogen bond results in the formation of a five-membered ring B (C3-C5/Br/H3B), which adopts envelope conformation with hydrogen atom displaced by -0.486 (3) Å from the plane of the other ring atoms.

In the crystal structure, intermolecular C-H···O [C2-H2A = 0.97, H2A···O1 = 2.32, C2···O1 = 3.193 (8) Å and C2-H2A···O1 = 150°] hydrogen bonds link the molecules (Fig. 2), in which they may be effective in the stabilization of the structure.

### Experimental

For the preparation of the title compound, beta-(2-bromo-4,5-dimethoxypenyl) -alpha-cyanoproponic acid (16 mmol) was dissolved in dimethylacetamide (10 ml), the mixture was heated at 443 K and evolution of the calculated amount of CO_2_ ceased after 30 min. The mixture was poured into water and set aside overnight. Crystals were separated, collected and washed with water and hexane. Crystals of (I) suitable for X-ray analysis were obtained by slow evaporation of a methanol solution.

### Refinement

H atoms were positioned geometrically, with C-H = 0.93, 0.97 and 0.96 Å for aromatic, methylene and methyl H, respectively, and constrained to ride on their parent atoms with U_iso_(H) = xU_eq_(C), where x = 1.5 for methyl H, and x = 1.2 for all other H atoms.

### Crystal data

**Table:**

C_11_H_12_BrNO_2_	*Z* = 8
*M**_r_* = 270.13	*F*_000_ = 1088
Tetragonal, *P*4_2_*b**c*	*D*_x_ = 1.556 Mg m^−^^3^
Hall symbol: P 4c -2ab	Mo *K*α radiation λ = 0.71073 Å
*a* = 17.552 (3) Å	Cell parameters from 25 reflections
*b* = 17.552 (3) Å	θ = 10–13º
*c* = 7.4870 (15) Å	µ = 3.54 mm^−^^1^
α = 90º	*T* = 294 (2) K
β = 90º	Block, colorless
γ = 90º	0.30 × 0.10 × 0.10 mm
*V* = 2306.5 (7) Å^3^	

### Data collection

**Table:**

Enraf–Nonius CAD-4 diffractometer	*R*_int_ = 0.047
Radiation source: fine-focus sealed tube	θ_max_ = 25.2º
Monochromator: graphite	θ_min_ = 1.6º
*T* = 294(2) K	*h* = −21→21
ω/2θ scans	*k* = −21→0
Absorption correction: ψ scan(North *et al.*, 1968)	*l* = 0→8
*T*_min_ = 0.416, *T*_max_ = 0.718	3 standard reflections
4281 measured reflections	every 120 min
1128 independent reflections	intensity decay: none
657 reflections with *I* > 2σ(*I*)	

### Refinement

**Table:**

Refinement on *F*^2^	H-atom parameters constrained
Least-squares matrix: full	*w* = 1/[σ^2^(*F*_o_^2^) + (0.052*P*)^2^] where *P* = (*F*_o_^2^ + 2*F*_c_^2^)/3
*R*[*F*^2^ > 2σ(*F*^2^)] = 0.052	(Δ/σ)_max_ < 0.001
*wR*(*F*^2^) = 0.113	Δρ_max_ = 0.36 e Å^−^^3^
*S* = 0.99	Δρ_min_ = −0.39 e Å^−^^3^
1128 reflections	Extinction correction: SHELXL97 (Sheldrick, 2008), Fc^*^=kFc[1+0.001xFc^2^λ^3^/sin(2θ)]^-1/4^
137 parameters	Extinction coefficient: 0.0026 (5)
Primary atom site location: structure-invariant direct methods	Absolute structure: Flack (1983), with no Friedel pairs
Secondary atom site location: difference Fourier map	Flack parameter: 0.00 (3)
Hydrogen site location: inferred from neighbouring sites	

### Special details

**Table:**

Geometry. All e.s.d.'s (except the e.s.d. in the dihedral angle between two l.s. planes) are estimated using the full covariance matrix. The cell e.s.d.'s are taken into account individually in the estimation of e.s.d.'s in distances, angles and torsion angles; correlations between e.s.d.'s in cell parameters are only used when they are defined by crystal symmetry. An approximate (isotropic) treatment of cell e.s.d.'s is used for estimating e.s.d.'s involving l.s. planes.
Refinement. Refinement of F^2^ against ALL reflections. The weighted R-factor wR and goodness of fit S are based on F^2^, conventional R-factors R are based on F, with F set to zero for negative F^2^. The threshold expression of F^2^ > 2sigma(F^2^) is used only for calculating R-factors(gt) etc. and is not relevant to the choice of reflections for refinement. R-factors based on F^2^ are statistically about twice as large as those based on F, and R- factors based on ALL data will be even larger.

### Fractional atomic coordinates and isotropic or equivalent isotropic displacement parameters (Å^2^)

**Table:**

	*x*	*y*	*z*	*U*_iso_*/*U*_eq_	
Br	0.95483 (5)	0.91044 (5)	0.9098 (3)	0.0796 (5)	
N	0.6404 (5)	0.9114 (5)	0.6468 (17)	0.083 (4)	
O1	0.7337 (3)	0.6500 (3)	0.8860 (13)	0.051 (2)	
O2	0.8737 (4)	0.6253 (3)	0.8147 (10)	0.055 (2)	
C1	0.6900 (6)	0.9379 (5)	0.7195 (18)	0.057 (3)	
C2	0.7527 (6)	0.9707 (5)	0.8211 (15)	0.060 (4)	
H2A	0.7383	1.0216	0.8585	0.073*	
H2B	0.7965	0.9754	0.7429	0.073*	
C3	0.7763 (5)	0.9255 (5)	0.9855 (13)	0.050 (3)	
H3A	0.7329	0.9212	1.0649	0.060*	
H3B	0.8158	0.9535	1.0482	0.060*	
C4	0.8058 (4)	0.8459 (4)	0.9433 (19)	0.040 (3)	
C5	0.8811 (4)	0.8304 (4)	0.906 (2)	0.043 (2)	
C6	0.9068 (5)	0.7583 (5)	0.8663 (14)	0.053 (5)	
H6A	0.9585	0.7501	0.8462	0.064*	
C7	0.8568 (5)	0.6988 (4)	0.8560 (11)	0.034 (3)	
C8	0.7797 (4)	0.7129 (4)	0.899 (2)	0.038 (2)	
C9	0.7556 (4)	0.7846 (4)	0.939 (2)	0.040 (3)	
H9A	0.7043	0.7929	0.9639	0.047*	
C10	0.6556 (4)	0.6598 (4)	0.932 (3)	0.056 (3)	
H10A	0.6297	0.6117	0.9225	0.084*	
H10B	0.6326	0.6957	0.8516	0.084*	
H10C	0.6519	0.6784	1.0520	0.084*	
C11	0.9502 (5)	0.6090 (5)	0.758 (2)	0.077 (4)	
H11A	0.9549	0.5556	0.7324	0.116*	
H11B	0.9852	0.6228	0.8507	0.116*	
H11C	0.9615	0.6378	0.6519	0.116*	

### Atomic displacement parameters (Å^2^)

**Table:**

	*U*^11^	*U*^22^	*U*^33^	*U*^12^	*U*^13^	*U*^23^
Br	0.0517 (6)	0.0477 (6)	0.1394 (12)	−0.0149 (4)	−0.0013 (13)	−0.0086 (14)
N	0.084 (7)	0.070 (6)	0.095 (10)	0.022 (5)	−0.025 (8)	−0.018 (7)
O1	0.039 (3)	0.036 (3)	0.078 (7)	−0.003 (2)	0.006 (5)	−0.015 (5)
O2	0.044 (4)	0.031 (4)	0.090 (6)	0.010 (3)	0.009 (4)	0.000 (4)
C1	0.067 (8)	0.041 (6)	0.062 (10)	0.013 (6)	0.002 (7)	−0.009 (6)
C2	0.064 (7)	0.032 (6)	0.085 (10)	0.008 (5)	−0.007 (7)	−0.010 (6)
C3	0.042 (6)	0.055 (6)	0.052 (9)	0.002 (5)	0.009 (5)	−0.012 (6)
C4	0.041 (5)	0.035 (4)	0.045 (8)	−0.001 (3)	−0.002 (7)	−0.006 (7)
C5	0.042 (5)	0.038 (5)	0.047 (7)	−0.011 (4)	−0.006 (9)	−0.007 (9)
C6	0.033 (4)	0.042 (5)	0.085 (14)	0.001 (4)	0.003 (6)	−0.003 (6)
C7	0.041 (5)	0.031 (4)	0.030 (8)	0.005 (4)	−0.008 (4)	−0.002 (4)
C8	0.039 (4)	0.026 (4)	0.048 (7)	−0.007 (3)	0.013 (8)	−0.001 (7)
C9	0.037 (4)	0.038 (4)	0.044 (7)	0.001 (4)	−0.005 (7)	0.002 (7)
C10	0.036 (5)	0.053 (5)	0.079 (8)	−0.013 (4)	0.001 (9)	0.008 (10)
C11	0.054 (6)	0.049 (6)	0.128 (13)	0.009 (5)	0.000 (8)	−0.013 (8)

### Geometric parameters (Å, °)

**Table:**

Br—C5	1.911 (7)	C4—C9	1.391 (10)
N—C1	1.128 (13)	C5—C6	1.376 (11)
O1—C8	1.371 (9)	C6—C7	1.367 (11)
O1—C10	1.422 (9)	C6—H6A	0.9300
O2—C7	1.358 (9)	C7—C8	1.414 (10)
O2—C11	1.438 (10)	C8—C9	1.360 (10)
C1—C2	1.456 (14)	C9—H9A	0.9300
C2—C3	1.521 (13)	C10—H10A	0.9600
C2—H2A	0.9700	C10—H10B	0.9600
C2—H2B	0.9700	C10—H10C	0.9600
C3—C4	1.524 (11)	C11—H11A	0.9600
C3—H3A	0.9700	C11—H11B	0.9600
C3—H3B	0.9700	C11—H11C	0.9600
C4—C5	1.376 (11)		
			
C8—O1—C10	116.9 (6)	C5—C6—H6A	119.9
C7—O2—C11	117.4 (7)	O2—C7—C6	126.7 (8)
N—C1—C2	177.3 (14)	O2—C7—C8	115.3 (7)
C1—C2—C3	115.0 (9)	C6—C7—C8	117.9 (8)
C1—C2—H2A	108.5	C9—C8—O1	125.3 (7)
C3—C2—H2A	108.5	C9—C8—C7	120.7 (7)
C1—C2—H2B	108.5	O1—C8—C7	113.9 (7)
C3—C2—H2B	108.5	C8—C9—C4	121.5 (8)
H2A—C2—H2B	107.5	C8—C9—H9A	119.2
C2—C3—C4	113.7 (8)	C4—C9—H9A	119.2
C2—C3—H3A	108.8	O1—C10—H10A	109.5
C4—C3—H3A	108.8	O1—C10—H10B	109.5
C2—C3—H3B	108.8	H10A—C10—H10B	109.5
C4—C3—H3B	108.8	O1—C10—H10C	109.5
H3A—C3—H3B	107.7	H10A—C10—H10C	109.5
C5—C4—C9	116.8 (7)	H10B—C10—H10C	109.5
C5—C4—C3	123.3 (7)	O2—C11—H11A	109.5
C9—C4—C3	119.9 (8)	O2—C11—H11B	109.5
C4—C5—C6	122.7 (7)	H11A—C11—H11B	109.5
C4—C5—Br	120.1 (6)	O2—C11—H11C	109.5
C6—C5—Br	117.1 (6)	H11A—C11—H11C	109.5
C7—C6—C5	120.2 (8)	H11B—C11—H11C	109.5
C7—C6—H6A	119.9		
			
C1—C2—C3—C4	62.4 (12)	C5—C6—C7—C8	−4.2 (18)
C2—C3—C4—C5	88.6 (16)	C10—O1—C8—C9	−6(3)
C2—C3—C4—C9	−90.8 (16)	C10—O1—C8—C7	178.6 (13)
C9—C4—C5—C6	0(2)	O2—C7—C8—C9	−178.5 (13)
C3—C4—C5—C6	−179.2 (13)	C6—C7—C8—C9	4(2)
C9—C4—C5—Br	−179.7 (11)	O2—C7—C8—O1	−2.4 (17)
C3—C4—C5—Br	1(2)	C6—C7—C8—O1	−179.8 (11)
C4—C5—C6—C7	2(2)	O1—C8—C9—C4	−177.5 (13)
Br—C5—C6—C7	−177.9 (9)	C7—C8—C9—C4	−2(3)
C11—O2—C7—C6	−7.0 (15)	C5—C4—C9—C8	0(3)
C11—O2—C7—C8	175.9 (12)	C3—C4—C9—C8	179.1 (13)
C5—C6—C7—O2	178.7 (11)		

### Hydrogen-bond geometry (Å, °)

**Table:**

*D*—H···*A*	*D*—H	H···*A*	*D*···*A*	*D*—H···*A*
C2—H2A···O1^i^	0.97	2.32	3.193 (10)	150
C3—H3B···Br	0.97	2.76	3.195 (9)	108

Symmetry codes: (i) −*x*+3/2, *y*+1/2, *z*.
